# Using large language models to assess public perceptions around glucagon-like peptide-1 receptor agonists on social media

**DOI:** 10.1038/s43856-024-00566-z

**Published:** 2024-07-10

**Authors:** Sulaiman Somani, Sneha S. Jain, Ashish Sarraju, Alexander T. Sandhu, Tina Hernandez-Boussard, Fatima Rodriguez

**Affiliations:** 1 Department of Medicine, Stanford University, Stanford, CA USA; 2 Division of Cardiovascular Medicine and the Cardiovascular Institute, Stanford University, Stanford, CA USA; 3 Department of Cardiovascular Medicine, Cleveland Clinic, Cleveland, OH USA; 4 Department of Biomedical Data Science, Stanford University, Stanford, CA USA

**Keywords:** Preventive medicine, Metabolic syndrome, Obesity

## Abstract

**Background:**

The prevalence of obesity has been increasing worldwide, with substantial implications for public health. Obesity is independently associated with cardiovascular morbidity and mortality and is estimated to cost the health system over $200 billion dollars annually. Glucagon-like peptide-1 receptor agonists (GLP-1 RAs) have emerged as a practice-changing therapy for weight loss and cardiovascular risk reduction independent of diabetes.

**Methods:**

We used large language models to augment our previously reported artificial intelligence-enabled topic modeling pipeline to analyze over 390,000 unique GLP-1 RA-related Reddit discussions.

**Results:**

We find high interest around GLP-1 RAs, with a total of 168 topics and 33 groups focused on the GLP-1 RA experience with weight loss, comparison of side effects between differing GLP-1 RAs and alternate therapies, issues with GLP-1 RA access and supply, and the positive psychological benefits of GLP-1 RAs and associated weight loss. Notably, public sentiment in these discussions was mostly neutral-to-positive.

**Conclusions:**

These findings have important implications for monitoring new side effects not captured in randomized control trials and understanding the public health challenge of drug shortages.

## Plain language summary

Obesity is a global public health burden that increases heart disease risk. Glucagon-like peptide-1 receptor agonists (GLP-1 RAs) are a class of medications originally developed for diabetes but are now used to improve lifespans in those with heart disease and increase weight loss. To better understand how the public views this type of drug, over 390,000 discussions from the social media platform Reddit were analyzed using computer software. Topics of discussion included experiences with weight loss, side effects of different GLP-1 RAs, and concerns about drug access and supply. The results showed a mainly neutral-to-positive view of these medications. The findings may help identify new side effects not previously seen in clinical trials and highlight future directions for research and public health efforts.

## Abstract (web summary)

Somani, Jain et al. evaluate public perceptions of glucagon-like peptide-1 receptor agonists with large language model-based analysis of social media posts. Sentiments were neutral-to-positive, and discussions included use in weight loss, side effects, and issues with access and supply.

## Introduction

Over 38% of the global population is currently overweight or obese, a number predicted to grow to 51% by 2035^1^. Obesity carries a fivefold associated risk of development of cardiometabolic disease (e.g., coronary artery disease, stroke, type II diabetes mellitus)^2^. As a result, obesity poses a ~30% increased risk for all-cause death for every five kg/m^2^ increase in body mass index (BMI) and remains an unmet area of clinical and public health need^3^.

Glucagon-like peptide-1 receptor agonizts (GLP-1 RAs) are a class of medications that mimic the action of endogenous GLP-1, an intestinal hormone that regulates glucose metabolism and satiety. These drugs were initially approved for the treatment of type 2 diabetes but have recently gained international attention for weight loss and cardiovascular risk reduction in obese patients with or without type II diabetes^4,5^. However, public perceptions of GLP-1 RAs, which can affect intended treatment uptake, access, and adherence are not well studied in the literature.

Social media platforms like Reddit provide a forum for anonymized public discourse on health topics and may identify real-world experiences not captured in clinical settings or randomized trials^6^. Manual analysis of large volumes of social media content to identify relevant topics of discussion is resource and time-intensive but may be accelerated using techniques in natural language processing, especially large language models (LLMs). We previously reported our artificial intelligence (AI) enabled topic modeling pipeline, composed of a series of natural language processing and unsupervised learning techniques, to analyze discussions on Reddit around statins and coronary artery calcium. In this study, we used LLMs to augment this approach for characterizing public perceptions about GLP-1 RAs on Reddit. We uncover topics around weight loss, side effects of different GLP-1 RAs, and concerns about drug access and supply, with a mainly neutral-to-positive view of GLP-1 RAs, highlighting the role of an AI-enabled pipeline to help monitor for emerging side effects, uncover public sentiment, and guide future directions for research and public health efforts.

## Methods

### Dataset curation

Reddit is a popular social media platform that is composed of communities called ‘subreddits’, which are prefixed by “r/” and are focused on specific topics (e.g., r/AskDoctors and r/WorldNews). Subreddits contain discussions composed of threads (“posts”) and responses (“comments”). Most subreddits, including all posts and comments contained within them, are openly visible to the public without the need for a Reddit user account. We curate all GLP-1 RA-related discussions via an Application Programming Interface called PullPush that indexes and permits retrieval of all openly available Reddit content by searching for discussions containing the brand- and generic names of available GLP-1 RA drugs: ‘semaglutide’, ‘rybelsus’, ‘wegovy’, ‘ozempic’, ‘tirzepatide’, ‘mounjaro’, ‘liraglutide’, ‘saxenda’, ‘retatrutide’, ‘dulaglutide’, ‘trulicity’, ‘exenatide’, ‘bydureon’, ‘byetta’, ‘lixisenatide’, ‘adlyxin’. The Stanford University Institutional Review Board deemed this study exempt from ethical review and the requirement for informed consent because no human participants were involved.

### Topic modeling

Our AI-enabled topic modeling, which clusters discussions into topics and groups, involves a series of three key steps: embedding discussions into a numerical representation using an embedding model, reducing dimensionality to decrease the complexity of this embedding, and clustering this representation to identify emergent topics, largely similar to previous work^6^. Discussions were first embedded into a numerical representation using a pretrained, document-level Bidirectional Encoder Representations from Transformers (BERT)-like architecture model called Beijing Academy of Artificial Intelligence (BAAI) Generalized Embeddings (bge-base-en-v1.5)^7^, which is trained over an extensive text corpus on both supervised and unsupervised techniques to achieve state-of-the-art performance on the Massive Text Embedding Benchmark^8^. These embeddings were then simplified into a lower dimensional representation using the Uniform Mapping Approximation and Projection algorithm to improve clustering performance. We initialize both the number and centroid of clusters using a density-based clustering algorithm called Hierarchical Density-Based Spatial Clustering of Applications with Noise (HDBSCAN) first and fine-tune the assignment of discussions into the appropriate topics using KMeans clustering.

Each topic is then labeled using Llama2 (7B), a freely available large language model by Meta, whose family of models achieves state-of-the-art performance compared to other LLMs in a variety of domains^9,10^. We engineer prompts to generate topic labels by passing representative discussions (chosen based on the Euclidean distance to the assigned topic’s centroid), randomly sampled discussions from the topic, and an initial set of topic keywords generated using a Bag-of-Words representation (Supplementary Fig. 1).

Since topics may be intrinsically distinguished by other embedded features from the model (e.g., linguistic style, tone) rather than meaningful content, we cluster the cumulative term-frequency inverse-document frequency (c-TF-IDF) representation of these topics using Spectral Clustering to find overarching themes of discussion (“groups”). The number of groups was automatically determined based on optimizing the Silhouette coefficient, which is a mathematical measure of how similar discussions are within a cluster relative to how similar those discussions are to those in other clusters. Group labels were generated by providing prompts to Llama2 with relevant topic labels (Supplementary Fig. 1). Further details on topic modeling are discussed in Supplementary Methods and Supplementary Table 2.

### Sentiment analysis

A separate BERT-like model, the Robustly Optimized BERT Pretraining Approach (RoBERTa), pretrained on characterizing sentiments from social media posts, was used to classify sentiment^11^. The output comprised of three probabilities (continuous values between 0 and 1) assigning the likelihood that the input text would have a negative, neutral, or positive sentiment. Sentiment value (‘positive’, ‘negative’, or ‘neutral’) for that phrase was assigned by choosing the sentiment with the highest probability. Further details around sentiment analysis are discussed in Supplementary Methods.

### Reporting summary

Further information on research design is available in the Nature Portfolio Reporting Summary linked to this article.

## Results

**Fig. 1:**
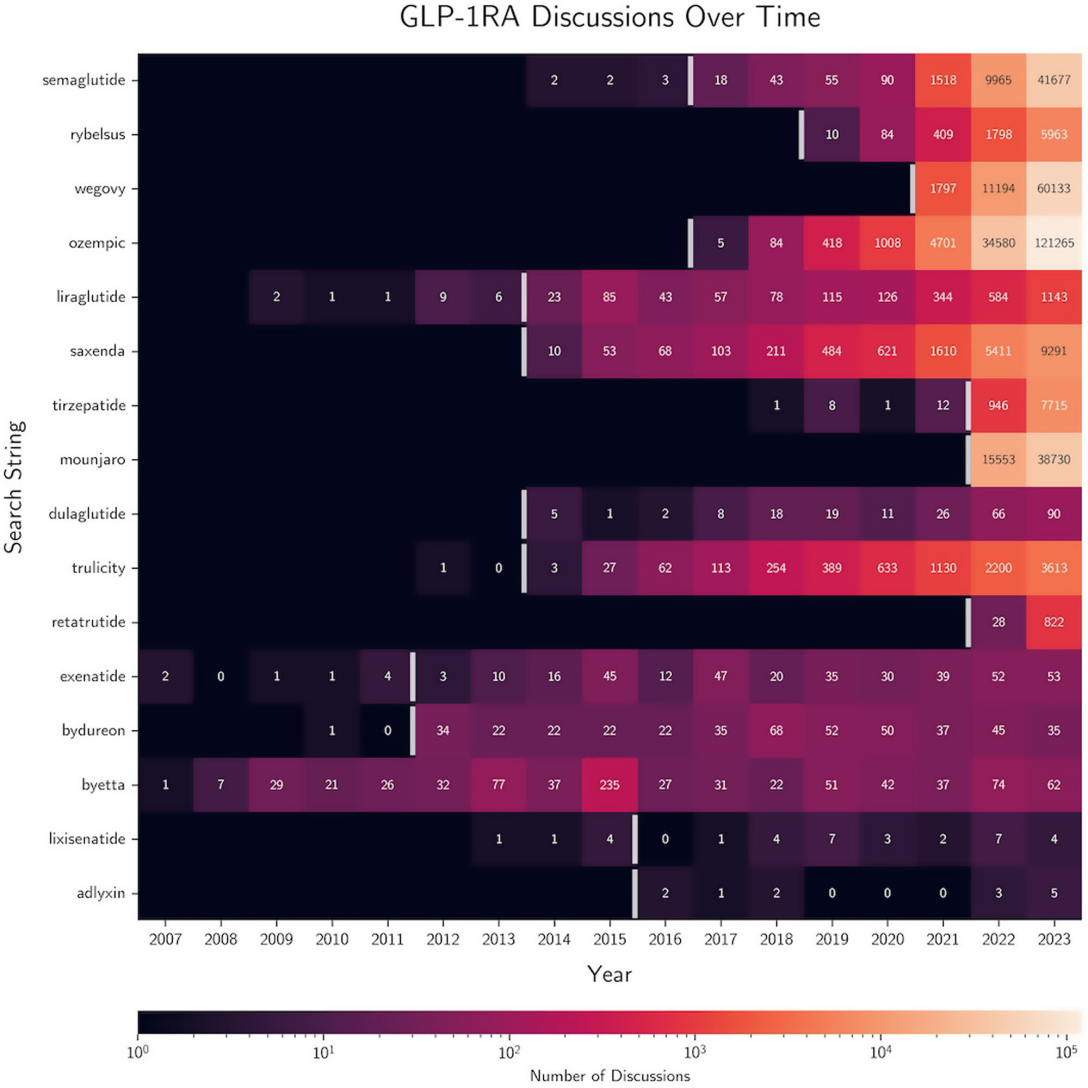
Glucagon-like peptide-1 receptor agonist discussions over time. Heatmap showing the number of glucagon-like peptide-1 receptor agonist (GLP-1 RA) discussions by the Reddit search string (generic name and all brand formulations) over time. Boxes are colored in logarithmic scale and annotated based on the number of discussions for that search string and year. Black boxes with no number indicate no Reddit posts prior to an initial post on the topic (any year with zero posts after the occurrence of an initial post is a black box labeled with a zero). White vertical lines depict a year of initial FDA approval for any clinical indication.

A total of 391,461 unique discussions, including 71,982 posts and 319,479 comments from 116,216 unique authors (Fig. 1), were included in our dataset. Most discussions (97.1%) focused on GLP-1 RA medications that are actively being prescribed for weight loss, including semaglutide, tirzepatide, and liraglutide. Across these brand- and generic drugs, ‘Ozempic’ had the most discussions (41.4%), even though it was not FDA-approved for weight loss in the United States. Only a minority of discussions (2.9%) focused on GLP-1 RAs that were only approved for use in diabetes mellitus. The number of discussions increased substantially over time, with greater than 95% of discussions taking place after January 1, 2022. Notably, this followed the 2021 Food and Drug Administration (FDA) approval for the brand formulation of semaglutide, Wegovy, which became the second GLP-1 RA to gain approval for weight loss.

Our topic modeling pipeline identified a total of 168 discussion topics (Supplementary Data 1), with most discussions focusing on individual experiences with GLP-1 RA for weight loss with respect to medication efficacy, comparison to other treatments, impact on appetite, and side effects. The most common side effect described, with respect to the total number of related topics, groups, and discussions, was nausea, followed by vomiting, constipation, injection site issues, pancreatitis, and gastroparesis; a more exhaustive list is provided in Supplementary Table 1. Some notable topics also included discussing access issues, including market shortages, ethics of off-label use, insurance coverage, and strategies to obtain GLP-1 RAs from nonprescription pharmacies internationally. Other identified topics reflected the positive impact of these medications for weight loss on motivation levels and mental health associated with specific obesity-related comorbidities (topics 64, 114). One topic also identified the value of these therapies in avoiding bariatric surgery (topic 76). Individuals described their experiences with different GLP-1 RA types and doses, celebrity endorsements, and side effects from dose adjustments. Of note, the LLM failed to assign topic labels to two topics (164, 168), citing that the content reflected within the prompt promoted illegal activities; on review, these discussions provided details on acquiring illicit substances, such as fentanyl and methamphetamine, in addition to formulations of semaglutide.

**Fig. 2:**
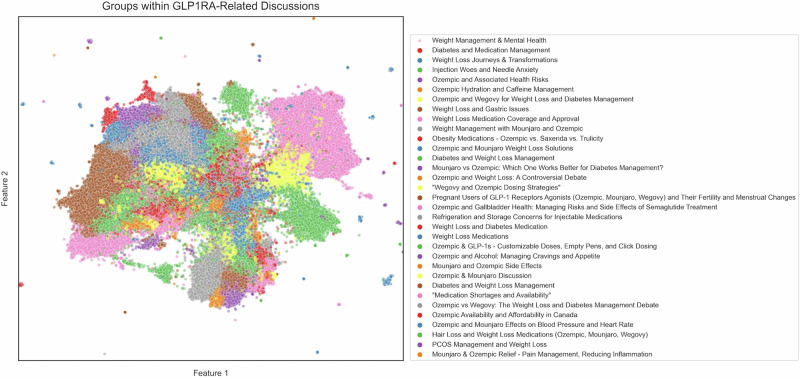
Topic modeling results. Scatter plot showing a 2D-projection of all discussion embeddings, where each point represents a discussion. The overlying color represents the associated group of that discussion based on the topic modeling. The *x*- and *y*-axes represent the two axes (Feature 1, Feature 2) onto which embeddings were dimensionally reduced using Uniform Manifold Approximation and Projection for visualization purposes.

Topics were further clustered into groups to find overarching themes of discussion (Fig. 2). A total of 33 groups were identified by maximizing the Silhouette coefficient (score 0.93, Methods). Most groups reflected the following four themes: (1) comparisons of weight loss efficacy with other treatments, supplements, and different GLP-1 RAs; (2) side effects and injectable administration nuances of GLP-1 RA; (3) access concerns including market availability and insurance coverage; and (4) anecdotal experiences of the positive psychological impact of GLP-1 RA use.

Finally, we performed a sentiment analysis on all discussions using a separate, pretrained language model was used to classify sentiment (i.e., “positive”, “neutral”, or “negative”). Of all discussions, 124,667 had negative (31.8%), 198,535 had neutral (50.1%), and 68,259 had positive (17.4%) sentiment.

## Discussion

In this qualitative study, we provide a dataset of 319,461 GLP-1 RA-related discussions from 116,216 different authors, with a notable increase in the number of discussions after regulatory approval of semaglutide for weight loss in 2021. Using an LLM-driven topic modeling pipeline, we characterize public perceptions about GLP-1 RAs from these discussions, uncovering a total of 168 discussion topics and 33 groups that focused on weight loss, comparison of side effects between GLP-1 RAs and with other medications/supplements, access issues, and the positive psychological benefit of GLP-1 RA. Sentiment analysis revealed a predominantly neutral-to-positive tone. These findings highlight the high public interest in GLP-1 RA and identify potential public health interventions for addressing obesity and cardiometabolic disease.

Our findings have several implications for the expanding role of GLP-1 RAs in the management of obesity and cardiometabolic disease that underscore the need to understand patient perceptions to guide clinical decision-making, research, and policy efforts. First, we uncover high public interest in GLP-1 RA, as demonstrated by the large volume of recent discussions, which surpasses our prior analysis of statins by an order of magnitude^6^. The most discussed GLP-1 RA was Ozempic, and most topics suggest its discourse lay in the context of weight loss. Coupled with the overall rise in GLP-1 RA-related discussions after the 2021 FDA approval of semaglutide for weight loss, we identify a high public interest in the use of these medications for weight management, despite their original intended use in diabetes. Additionally, while clinical guidelines for indicated use of GLP-1 RAs are rapidly changing as evidence for GLP-1 RA use evolves, the ethics of off-label use are particularly important to consider given the impact these shortages can have on access for patients who otherwise have a strong clinical indication to be on them. In the wake of the recent Wegovy shortage, our pipeline also identifies concerning discussions around obtaining GLP-1 RAs internationally (e.g., Mexico or Canada) through nonprescription pharmacies and online black markets where other illicit substances could be obtained. This finding is alarming given the quality control and regulation issues surrounding these routes and poses a unique public health threat. These public behaviors highlight the value of capturing social media discussions and our pipeline to identify them.

Second, we identify many topics and groups focused on medication side effects. Many of the discussions on the topics of side effects include associations that have been reported in meta-analyses and randomized controlled trials (e.g., nausea, vomiting, diarrhea, injection site reactions, pancreatitis, and gastroparesis)^12–15^. However, other discussions focus on side effects that are otherwise not well established (e.g., menstrual cycle changes, increased fertility, depression, anxiety, flu vaccine sensitivities, myalgias), which merit further study. Currently, the FDA provides an Adverse Event Reporting System to monitor post-market approval medication side effects. Identifying drug side effects could complement the phase IV medication data collected through the FDA and create a novel strategy to leverage social media data to uncover potential new side effects or a higher frequency of previously described side effects. Utilizing the pipeline above could efficiently monitor for other emerging, patient-reported experiences and adverse events over time.

Third, our study reveals mostly neutral-to-positive sentiment for these drugs, which is in strong contrast with prior analyses showing a predominantly negative-to-neutral tone toward other commonly prescribed cardiovascular medications (e.g., statins)^6^. Topics and groups emphasized the positive impact of GLP-1 RAs and the impact successful weight loss has had on their motivational levels and overall mental health. Although patients may regain nearly two-thirds of their lost weight on GLP-1 RAs after cessation of the drug^16^, this early positive reinforcement may serve as an incentive to pursue a long-term, multipronged approach to combat obesity.

This study should be interpreted in the context of several limitations, including (1) the impact of spelling errors on discussion mislabeling, (2) the inability to adjudicate reported side effects, (3) the demographic characteristics of Reddit authors which may limit generalizability, and (4) use of pre-existing general task benchmarks to drive LLM choice that may not optimally generalize to the topic labeling task.

In conclusion, we provided a dataset of nearly 400,000 GLP-1 RA-related discussions, most of which were posted after regulatory approval of semaglutide for weight loss in 2021. Using an LLM-driven topic modeling pipeline, we discovered that discussions focused on the use of GLP-1 RAs for weight loss, the comparison of side effects between GLP-1 RAs and with other medications/supplements, access issues, and the positive psychological benefit of GLP-1 RA and associated weight loss. Together, these findings suggested high public interest in GLP-1 RA and identified potential priorities for the clinical and policy communities, including monitoring side effects, addressing access barriers, and acknowledging both the physical and psychological benefits of GLP-1 RAs.

### Supplementary information

Supplementary Information

Description of Additional Supplementary Files

Supplementary Data 1

Reporting Summary

## Data Availability

The dataset of all Reddit discussions used in this study and source data for Figs. 1 and 2 can be freely accessed at 10.5281/zenodo.12209343^17^.

## Code availability

All code developed for data collection, topic modeling, and sentiment analysis is made freely available on 10.5281/zenodo.12209343^17^.
