# Supplementary material for: Using large language models to assess public perceptions around glucagon-like peptide-1 receptor agonists on social media
Source: Commun Med (Lond). 2024 Jul 10;4:137. doi: 10.1038/s43856-024-00566-z (PMC11237093; doi:10.1038/s43856-024-00566-z)
Supplement: Supplementary file 1 — Supplementary Information [file 43856_2024_566_MOESM1_ESM.pdf]

Using Large Language Models to Assess Public Perceptions Around Glucagon-Like Peptide-1 Receptor Agonists on Social Media

Authors: Sulaiman Somani*, Sneha S. Jain*, Ashish Sarraju, Alexander T. Sandhu, Tina Hernandez-Boussard, Fatima Rodriguez

*these authors contributed equally to this work

Supplementary Information

Supplementary Methods 2
Supplementary Figure 1 4
Supplementary Table 1 5
Supplementary Table 2 6
Supplementary References 7

Supplementary Methods

Preprocessing

To prepare the raw text scraped from Reddit for topic modeling, the following series of operations are performed. Since every post is composed of both a title and body, these two attributes are concatenated and separated by a period. Next, hyperlinks and html tags from the body of each discussion are removed to reduce content size and reduce the risk of erroneous embeddings during topic modeling. Discussions that were less than five characters in length were also removed.

Topic Modeling

Topic modeling is a use case of natural language processing that identifies clusters (or topics) of similar information from a set of text documents. Bidirectional Encoder Representations from Transformers (BERT) models are a state-of-the-art architecture that have generally outperformed prior models in embedding words, sentences, and documents with greater contextual and semantic meaning. Notably, the foundational transformer block in BERT models is also a crucial component of large language models (LLM) and state-of-the-art computer vision models, highlighting the valuable role of this novel architectural design in modern artificial intelligence models.

We first start embedding documents using a pretrained, document-level Bidirectional Encoder Representations from Transformers (BERT)-like architecture model called Beijing Academy of Artificial Intelligence (BAAI) Generalized Embeddings (bge-base-en-v1.5), which is a 102M parameter model that uses the final layer's hidden state (768 dimensions) of the [CLS] (sentence classification prediction) token as the embedding representation.[1] This class of embedding models achieves state-of-the-art performance on the Massive Text Embedding Benchmark, which is an evaluation pipeline that can be used to assess performance of embedding models across 8 embedding tasks (including clustering tasks) using 58 different datasets.[2]

Clustering discussions with a large feature size (i.e., 768 as the output dimensionality of the embedding) can be computationally prohibitive; as such, we further reduce the dimensionality of this representation using Uniform Manifold Approximation and Projection (UMAP), an unsupervised dimensionality reduction algorithm that better preserves global topology of higher-dimensional data into lower dimensions.[3] We then cluster these discussions into relevant topics in a series of two steps. First, we find an initial guess of clusters of documents corresponding to a particular topic using Hierarchical Density Based Spatial Clustering of Applications with Noise (HDBSCAN), which chooses the optimal number of clusters based on techniques in graph theory to assess for cluster stability.[4] However, this technique carries a risk of excessively labeling points as outliers for topics that may be smaller in size and miss out on labeling topics that are otherwise relevant to captures. For this reason, we use the initial number and locations of clusters obtained from HDBSCAN as an initial guess for a less stringent and more commonly used clustering algorithm, KMeans Clustering. After topics are identified, we use a class-based term-frequency inverse-document frequency (c-TF-IDF) technique, similar to a Bag-of-Words representation, based on the discussions contained in each topic to identify keyword representations of each topic.

To create topic labels that can better represent a topic, we leverage LLMs and engineer prompts for this task, as shown in **Supplementary Figure 1**. We choose Llama2 (7B) as our LLM of choice, given its free availability, transparent development, and state-of-the-art performance of its family of models compared to other LLMs in a variety of domains in the Holistic Evaluation of Language Models (HELM) benchmark.[5,6] Unique prompts for each topic are created with a context ("You are a honest, scientific chatbot that helps me, a Cardiologist, create unique, diverse labels for a topic based on representative discussions and keywords."), constraint instructions ("Do not be creative or loquacious. Please present the topic label in a short and direct manner."), input data ("I have a topic that is described by the following keywords:<keywords>…In this topic, the following documents are a small but representative subset of all other documents in the topic…"), and output instruction ("Based on the information above, can you create three short, direct labels without descriptions for this topic?"). Our input data consists of five representative discussions, five random discussions, and keyword representations of each topic. Representative discussions are chosen as those discussions closest in Euclidean distance in the native embedding space to the centroid (represented as the mean of all embedded

discussions in that topic) of that topic. Five randomly sampled discussions from within the topic, without pre-specifying that these are random to the LLM, are also provided to capture the variety of discussions that may be captured in each topic.

Since these topics may be granular, identifying further clusters within these topics is important to provide an appreciation of the overarching themes within our dataset. To group these topics, we use UMAP and spectral clustering[7] on the c-TF-IDF representation of each topic. Since spectral clustering requires a prespecified number of clusters to be provided, a sensitivity analysis was performed by measuring the Silhouette Coefficient across a range of prespecified clusters (2 to 40) to find the optimal number of clusters.[8,9] The Silhouette Coefficient measures how the overlap and mislabeling across clusters by calculating the mean intra-cluster distance and the mean nearest-cluster distance. Values range between -1, which suggests likely misassignment of a discussion sample to an incorrect cluster, and 1, which suggests optimum assignment of a discussion point to a cluster; a value of zero indicates overlapping clusters. Labels for the resulting groups are generated by using Llama2 and a prompt similar in context and instructions as before using the topic labels for all topics within that group, as outlined in **Supplementary Figure 1**.

All analysis above was performed using Python 3.11. Hyperparameters for the above models are specified in **Supplementary Table 2**. All models with built in stochasticity were initialized with a random state set to 42 to enable reproducibility. All code is publicly available on www.github.com/sssomani/glp1_reddit.

<u>Sentiment Analysis</u>

Sentiment analysis is a form of natural language processing that classifies the sentiment of text documents into distinct categories, most commonly “positive” (“I am so happy about the weight I lost on Ozempic!”) or negative (“I had the worst nausea and vomiting on Ozempic!”). Multiclass models have also been developed that additionally classify documents into a neutral category when these text representations may not be polarized towards a positive or negative sentiment. For example, the phrase “tirzepatide is a drug” is not opinionated and as such does not fit the mold of most traditional sentiment analysis models. Emerging techniques for sentiment analysis leverage the same transformer model architecture, with pretrained models available on open source frameworks like Huggingface.[10] To assess sentiments for each post, a separate BERT-like model, Robustly Optimized BERT Pretraining Approach (RoBERTa), pretrained on characterizing sentiments from social media posts, was used to classify sentiment.[11] This model is useful since it offers multiclass labels (i.e. “positive”, “neutral’, or “negative” classification of text) and has been used in recent literature investigating healthcare problems using data from social media.[12–15] The length of the input phrase was limited to 512 characters for this model. The output comprised of three probabilities assigning the likelihood that the input text would have a negative, neutral, or positive sentiment. For phrases less than or equal to 512 characters, the preprocessed text was passed with assignment of the sentiment with the highest probability to that phrase; for instance, if a phrase had the following probability array (0.1, 0.3, 0.6) for (negative, neutral, positive), then that phrase would be labeled as ‘positive’. To process longer phrases, multiple phrases from each post or comment exceeding 512 characters were created by searching for all instances of any of the search words (e.g. “ozempic”) and taking the 256 characters before and after that match location. Sentiment value (‘positive’, ‘negative’, or ‘neutral’) for that phrase was assigned by taking the mean of the probabilities across all subsampled regions and choosing the sentiment with the highest probability. To understand how sentiments varied across topics and groups, the sentiment label was transformed from ‘negative’, ‘neutral’, and ‘positive’ to -1, 0, and 1, respectively.

Supplementary Figure 1. AI Topic Modeling Pipeline. The topic modeling pipeline in this study is described. First, candidate discussions about GLP-1RA are extracted from all Reddit discussions using candidate key terms and preprocessed for our AI models (“Dataset Curation”, red box). Next, each discussion is embedded, dimensionally reduced, and clustered using KMeans to identify salient topics (“Topic Identification,” yellow box), with subsequent identification of groups based on clustering each topic’s cumulative term-frequency inverse-document frequency representation (“Group Identification,” blue box). Topic labels for both topics and groups are generated using a large language model, Llama, with the specified prompt template (“Topic and Group Labeling using Llama”, purple box). The topic labeling prompt template is shown in the center with a yellow highlight, while the group labeling prompt template is shown in blue on the right. Sentiment analysis is performed directly on the preprocessed set of discussions (“Sentiment Analysis”, green box).

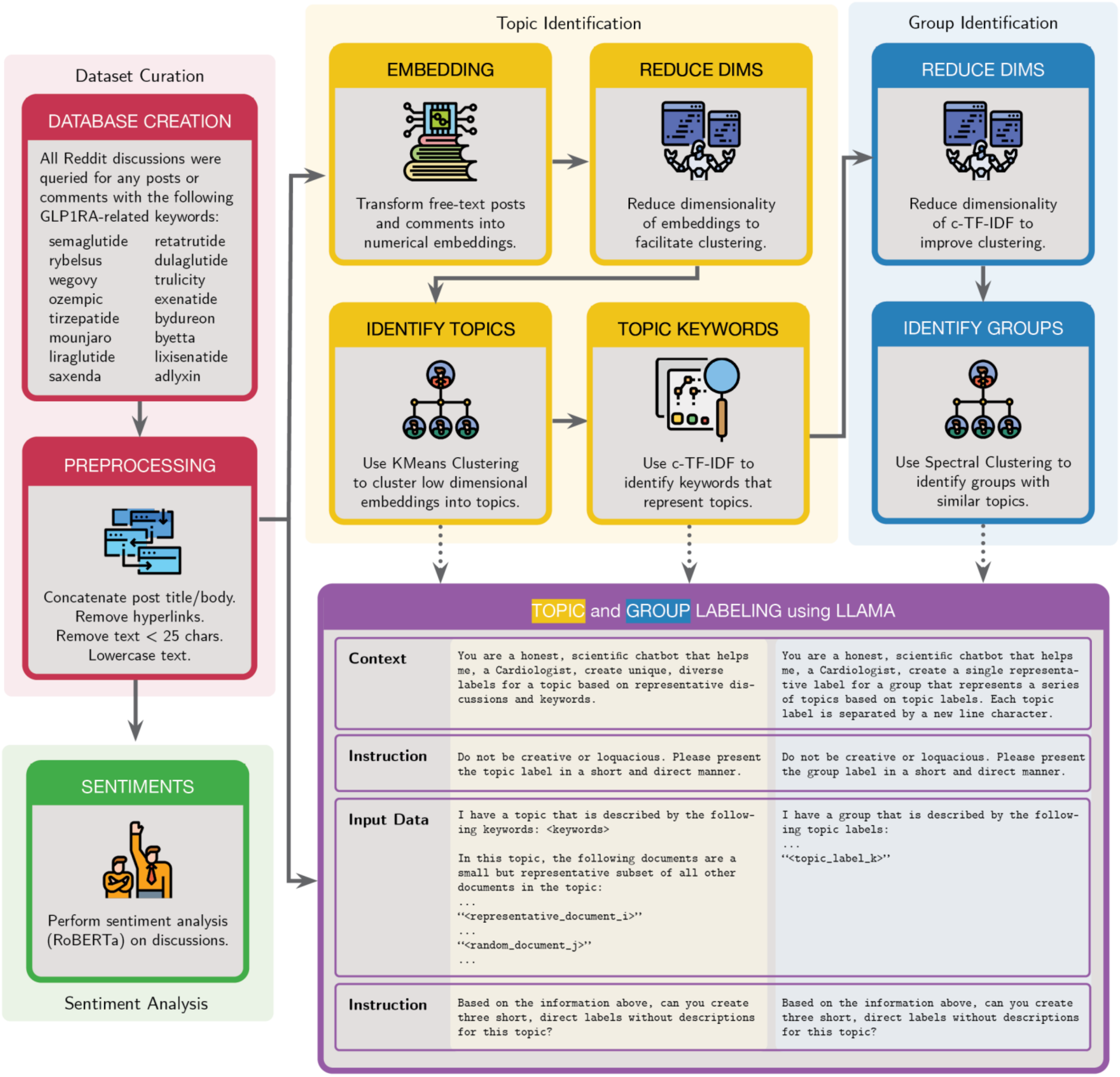

Supplementary Table 1: Notable Side Effects from Topic Modeling.

Side Effect	Topics	Known Side Effect of Semaglutide[16,17]	Known Side Effect of Liraglutide[18]	Know Side Effect of Tirzepatide[19,20]
General GI side effects (nausea, vomiting, constipation, diarrhea, abdominal pain, gastroparesis)	6, 11, 59, 65, 67, 82, 84,85,106, 112, 141	X	X	X
Lightheadedness, hypotension, or dehydration	96, 144, 145, 156	X	X	X
Thyroid disease or thyroid cancer risk	89, 120, 130	X	X	X
Pancreatitis	127, 138	X	X	X
Injection site issues	137, 152	X	X	X
Migraines/headaches	107, 118	X	X	X
Skin sensitivity or rashes	107, 148	X	X	X
Tachycardia	126, 157	X	X	X
Vision changes	126, 149	X*	X*	X*
Cholelithiasis	124	X	X	X
Kidney Injury	150	X	X	X
Fatigue	88, 96, 109	X		X
Hair loss	111, 155	X		X
Interaction with birth control pills	102			X
Depression, Anxiety, or increase in Suicidal thoughts	103, 105, 126, 133			
Menstrual cycle disturbances, increased fertility, and decreased polycystic ovarian syndrome symptoms	40, 47, 75, 154			
Facial changes of weight loss	41, 42			
Aversion to alcohol	73, 115			
Myalgias	132			
Vaccine sensitivities	161			

Analysis based on FDA full prescribing information for current FDA-approved GLP-1RAs for obesity and a formulation of semaglutide that is commonly prescribed for weight loss but not currently FDA approved.
*Blurred vision listed in full prescribing information as a sign of possible hypoglycemia.

Supplementary Table 2: Hyperparameters for Topic Modeling.

	**Hyperparameter**	**Value**
**UMAP (Topics)**	Number of neighbors	15
	Number of dimensions (output)	10
	Distance metric	‘cosine’
	Spread	1.0
	Learning rate	1.0
	Minimum distance between embedded points	0.0
**HDBSCAN**	Minimum cluster size	100
	Metric	‘euclidean’
**UMAP (Groups)**	Number of neighbors	2
	Number of dimensions (output)	3
	Distance metric	‘hellinger’
	Spread	1.0
	Learning rate	1.0
	Minimum distance between embedded points	0.1
**Spectral Clustering (Groups)**	Number of components	2

Supplementary References

1. Xiao, S., Liu, Z., Zhang, P. & Muennighof, N. C-Pack: Packaged resources to advance general Chinese embedding. *arXiv [cs.CL]* (2023).

2. Muennighoff, N., Tazi, N., Magne, L. & Reimers, N. MTEB: Massive Text Embedding Benchmark. *arXiv [cs.CL]* (2022).

3. McInnes, L., Healy, J. & Melville, J. UMAP: Uniform Manifold Approximation and Projection for Dimension Reduction. *arXiv [stat.ML]* (2018).

4. Campello, R. J. G. B., Moulavi, D., Zimek, A. & Sander, J. Hierarchical density estimates for data clustering, visualization, and outlier detection. *ACM Trans. Knowl. Discov. Data* **10**, 1–51 (2015).

5. Liang, P. *et al.* Holistic Evaluation of Language Models. *arXiv [cs.CL]* (2022).

6. Touvron, H. *et al.* Llama 2: Open foundation and fine-tuned chat models. *arXiv [cs.CL]* (2023).

7. von Luxburg, U. A Tutorial on Spectral Clustering. *arXiv [cs.DS]* (2007).

8. Rousseeuw, P. J. Silhouettes: A graphical aid to the interpretation and validation of cluster analysis. *J. Comput. Appl. Math.* **20**, 53–65 (1987).

9. Davies, D. L. & Bouldin, D. W. A Cluster Separation Measure. *IEEE Trans. Pattern Anal. Mach. Intell.* **PAMI-1**, 224–227 (1979).

10. Models. https://huggingface.co/models.

11. Loureiro, D., Barbieri, F., Neves, L., Espinosa Anke, L. & Camacho-collados, J. TimeLMs: Diachronic language models from twitter. in *Proceedings of the 60th Annual Meeting of the Association for Computational Linguistics: System Demonstrations* (Association for Computational Linguistics, Stroudsburg, PA, USA, 2022). doi:10.18653/v1/2022.acl-demo.25.

12. Kolluri, N., Liu, Y. & Murthy, D. COVID-19 misinformation detection: Machine-learned solutions to the infodemic. *JMIR Infodemiology* **2**, e38756 (2022).

13. Noraset, T., Chatrinan, K., Tawichsri, T., Thaipisutikul, T. & Tuarob, S. Language-agnostic deep learning framework for automatic monitoring of population-level mental health from social networks. *J. Biomed. Inform.* **133**, 104145 (2022).

14. Baker, W. *et al.* Classification of Twitter vaping discourse using BERTweet: Comparative deep learning study. *JMIR Med. Inform.* **10**, e33678 (2022).

15. Anetta, K., Horak, A., Wojakowski, W., Wita, K. & Jadczyk, T. Deep learning analysis of Polish electronic health records for diagnosis prediction in patients with cardiovascular diseases. *J. Pers. Med.* **12**, 869 (2022).

16. WEGOVY Full Prescribing Information. https://www.accessdata.fda.gov/drugsatfda_docs/label/2023/215256s007lbl.pdf.

17. OZEMPIC Full Prescribing Information. https://www.accessdata.fda.gov/drugsatfda_docs/label/2017/209637lbl.pdf.

18. SAXENDA Full Prescribing Information. https://www.accessdata.fda.gov/drugsatfda_docs/label/2023/206321s016lbl.pdf.

19. MOUNJARO Full Prescrbing Information. https://www.accessdata.fda.gov/drugsatfda_docs/label/2022/215866s000lbl.pdf.

20. ZEPBOUND Full Prescribing Information. https://www.accessdata.fda.gov/drugsatfda_docs/label/2023/217806s000lbl.pdf.
