# Supplementary material for: Using large language models to assess public perceptions around glucagon-like peptide-1 receptor agonists on social media
Source: Commun Med (Lond). 2024 Jul 10;4:137. doi: 10.1038/s43856-024-00566-z (PMC11237093; doi:10.1038/s43856-024-00566-z)
Supplement: Supplementary file 2 — Description of Additional Supplementary Files [file 43856_2024_566_MOESM2_ESM.pdf]

Description of Additional Supplementary Files:

File name: Supplementary Data 1

File description: All topics uncovered in the topic modeling pipeline are described here, as well as the number of discussions (by posts and comments), the topic label generated by Llama, and the most representative discussion for each topic. Each representative discussion is chosen as the discussion whose location in the embedding space is closest, by Euclidean distance, to that topic's centroid (mean of all embedded discussions belonging to that topic). Authors of this manuscript only edited essential aspects; specifically, shortening posts to limit excessive length, replacing expletives with ***, and censoring one post as noted below. For posts in other languages, we provided an English translation using Google Translate.
